# Scalp-Ear-Nipple Syndrome: A Case Report

**DOI:** 10.1155/2014/785916

**Published:** 2014-02-09

**Authors:** Estela Morales-Peralta, Vivian Andrés, Dainé Campillo Betancourt

**Affiliations:** ^1^National Center of Medical Genetics, 146, No. 3102, Playa, 11600 La Habana, Cuba; ^2^Genetic Counselor, Center of Medical Genetics, Sibanicú Municipality, Camaguey, Cuba

## Abstract

The scalp-ear-nipple (SEN) syndrome is an infrequent congenital disease. Its main features are scalp defects, malformed ears, and absence of nipples. Most of the reported cases are autosomal dominant. We report on a patient suffering SEN syndrome with possible autosomal recessive inheritance. It is concluded that SEN syndrome should be recognized as an entity with genetic heterogeneity once there is evidence of different genetic manner of inheritance described in this disease.

In 1978 Finlay and Marks [1] described scalp defects, malformed ears, and absence of nipples in several members of a family over five generations. At the present time, this syndrome is well known as SEN syndrome. So far, only a few patients have been described, most of them with an autosomal dominant inheritance [2].

In 2007 Al-Gazali et al. [3] reported two children from an inbred Arab family who had symptoms of hypotonia and developmental delay in addition to the main features of SEN syndrome. They suggested a severe recessive form of this syndrome. We describe a SEN syndrome patient and we suggest a possible recessive inheritance.

The proband was a female born through spontaneous vaginal delivery at term following an uneventful pregnancy. Her parents were cousins and she had six siblings; two of the siblings, a male and female, had almost identical pattern of congenital defects (Figure 1(a)). This sister (IV.5) had renal agenesis and died of renal failure. Her affected brother suffered a ventricular septal defect and died because of heart failure. Other relatives, including both parents, were normal. Her psychomotor development was normal, so were all of their siblings.

On physical examination at 34 years old she had normal body measurements and thin sparse hair, scalp nodules, without hair over them, low nasal bridge, excess of nasofrontal soft tissue with dystopia canthorum, widely spaced teeth with oligodontia, cupped protruding ears with small tragus and antitragus, absent nipples, partial skin sindactilia of third and fourth fingers and second and third toes—with thin and hypoplastic nails—and camptodactyly of fifth fingers, dry skin, and scanty secondary sexual hair (Figure 1(b)).

The karyotype, established on peripheral blood cultures after GTG banding, was 46, XX. All laboratory remaining data were within the normal range, so were skull X-ray, abdominal ultrasound, and electrocardiogram.

This patient had all major clinical features described in SEN syndrome [4]. An autosomal recessive pattern of inheritance was evident in her family—all had consanguineous parents and it was present in both sexes. Another possible explanation that this disease appeared in more than one member of kindred would be germline mosaicism.

Al-Gazali et al. [3] had already suggested a recessive form of SEN syndrome in their description of two affected in an inbred family. Since their patients had severe hypotonia and developmental delay and these signs had not yet been noted in previously reported cases suffering SEN syndrome, they suggested that the disorder in these children may be a severe recessive form of SEN syndrome characterized by the presence of severe hypotonia and developmental delay. Neither the patient, nor her affected siblings, had these features. On the other hand, the children described by Al-Gazali et al. [3] did not have camptodactyly, syndactyly, and dry skin, features frequently seen in SEN syndrome [5]; however all of these clinical features were observed in our case. All differences observed between these patients could be explained by variable expressivity.

There is no specific laboratory test available for this syndrome; therefore, its diagnosis is primarily by its major clinical features of which all were present in this case as well in the children described by Al-Gazali et al. [3].

The main different diagnosis of SEN Syndrome must be done with other syndromes associated with the absence of nipples. They include ectodermal dysplasia with hydrosis and ectodermal dysplasia with anhydrosis. Aplasia cutis, another feature of SEN syndrome, has not been found in any of them [2].

Homozygous deficiency of lymphoid enhancer factor-1 (Lef-1) gen causes whiskers and hair in mice, absence of mammary glands, and edentulism. For this reason, van Steensel et al. [6] suggested that lymphoid enhancer factor-1 (Lef-1) might be a potential candidate gene for SEN syndrome. If homozygous deficiency is a well-known cause of recessive inheritance and it was reported as the cause of a congenital pattern in mice similar to SEN syndrome, this inheritance pattern could also be observed in human's SEN syndrome, especially when it occurs in inbred families. However, most patients suffering from described SEN syndrome had autosomal dominant inheritance. In conclusion, our observation suggests that SEN syndrome should be recognized as an entity with genetic heterogeneity once there is evidence of different genetic manner of inheritance described in this disease.

## Figures and Tables

**Figure 1:**
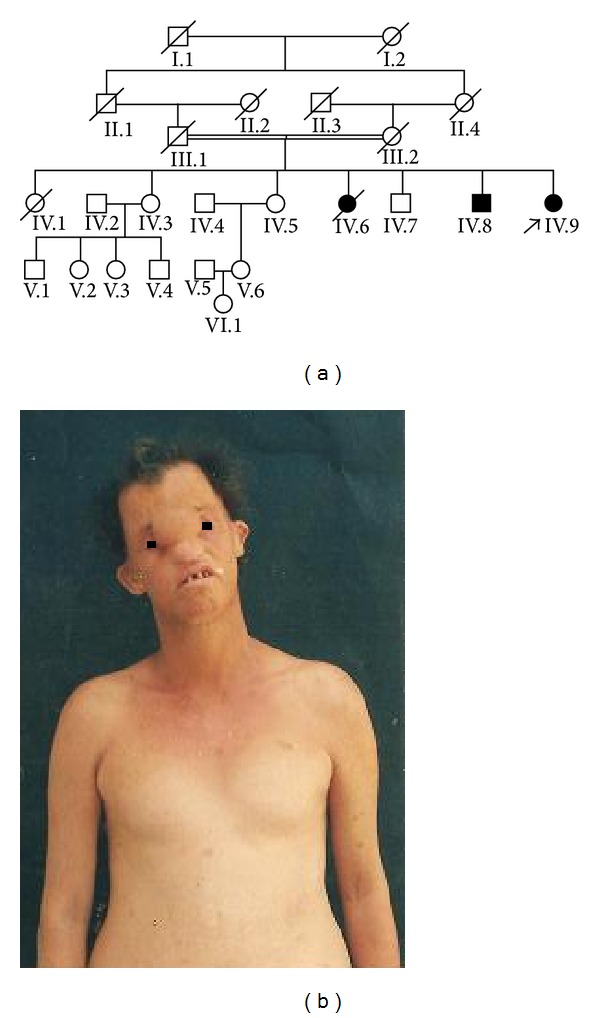
(a) Familiar tree. (b) The propositus: observe the following: excess of soft tissue on nasofrontal region, widely spaced teeth, cupped protruding ears, and absent nipples.
